# Automatic Method for Vickers Hardness Estimation by Image Processing

**DOI:** 10.3390/jimaging9010008

**Published:** 2022-12-30

**Authors:** Jonatan D. Polanco, Carlos Jacanamejoy-Jamioy, Claudia L. Mambuscay, Jeferson F. Piamba, Manuel G. Forero

**Affiliations:** 1 Semillero Lún, Grupo D+Tec, Faculty of Engineering, Universidad de Ibagué, Ibagué 730007, Colombia; 2 Semillero NOVAMAT, Faculty of Natural Science and Mathematics, Universidad de Ibagué, Ibagué 730007, Colombia; 3 Professional School of Systems Engineering, Faculty of Engineering, Architecture and Urban Planning, Universidad Señor de Sipán, Chiclayo 14000, Lambayeque, Peru

**Keywords:** Vickers hardness, hardness estimation, image processing, steel heat treating, mechanics of materials

## Abstract

Hardness is one of the most important mechanical properties of materials, since it is used to estimate their quality and to determine their suitability for a particular application. One method of determining quality is the Vickers hardness test, in which the resistance to plastic deformation at the surface of the material is measured after applying force with an indenter. The hardness is measured from the sample image, which is a tedious, time-consuming, and prone to human error procedure. Therefore, in this work, a new automatic method based on image processing techniques is proposed, allowing for obtaining results quickly and more accurately even with high irregularities in the indentation mark. For the development and validation of the method, a set of microscopy images of samples indented with applied forces of 5N and 10N on AISI D2 steel with and without quenching, tempering heat treatment and samples coated with titanium niobium nitride (TiNbN) was used. The proposed method was implemented as a plugin of the ImageJ program, allowing for obtaining reproducible Vickers hardness results in an average time of 2.05 seconds with an accuracy of 98.3% and a maximum error of 4.5% with respect to the values obtained manually, used as a golden standard.

## 1. Introduction

The study of the properties of materials is of great importance to determine their behavior in specific applications. Mechanical properties such as hardness, ductility, or stiffness can be studied from laboratory tests to determine the appropriate characteristics for their use. Hardness is one of the most important mechanical properties of materials, as it allows for determining the resistance to deformation by a harder material [1,2]. There are different hardness tests and these vary depending on the type of material, e.g., the Brinell hardness test is best suited to determine the hardness of wood-based materials, or materials with relatively low hardnesses [3]. On other hand, the Mohs hardness is the most commonly used to identify minerals [4], Shore hardness implemented in polymeric materials [5] and Vickers hardness to determine the hardness of metals, ceramics, and other materials. Studies have shown that modifications can be made to Vickers hardness equipment to determine other properties such as elasticity or surface stresses of the material [6,7].

Materials such as steel used in automotive axles, cutting tools, among other applications, are subjected to constant forces or loads, which can cause deformation or breakage of the material. The search for continuous improvement has led to the implementation of materials in the form of thin films, which consist of improving the surface properties of the substrate such as steel, providing high hardness, low coefficient of friction, and wear and corrosion resistance in chemically aggressive environments, allowing for increasing its useful life and covering a wide field of applications [8,9,10,11,12].

There are different methods to modify the hardness of the material, either by heat treatment such as quenching or tempering to modify the microstructure [11,12] or by depositing thin film coatings on the surface of the base material. Among the most commonly used coatings in the industry are Titanium Nitride (TiN) and Niobium Nitride (NbN). In the present work, D2 steel samples with different heat treatments and steels with Titanium Niobium Nitride (TiNbNx) coatings were used, which are employed for industrial applications as a cutting tool [13,14,15].

**Figure 1:**
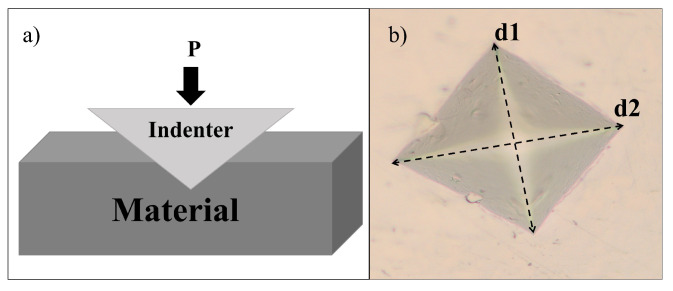
Indentation process of a material sample. (**a**) force applied with an indenter to the material; (**b**) indentation mark.

As mentioned above, one way to determine the quality of the material is from tests such as the Vickers hardness test (HV), allowing for estimating this property by measuring the plastic deformation or imprint produced on its surface after applying a force with a square-based, pyramid-shaped diamond indenter with face angles of 136${}^{\circ }$ (see Figure 1) [16]. Thus, once the test has been performed, the impression is visualized through an optical microscope and the indentation diagonals are measured to determine the Vickers hardness value. This type of test is widely used for quality control, as it allows for determining if a material is suitable for a given application. The equation for determining the Vickers hardness value is given by:

(1) $$HV=0.1891\frac{P(N)}{D^{2}\left({\mathrm{mm}}^{2}\right)}$$

where *D* represents the average diagonal distance (d1 and d2) of the indentation mark and P the force applied to the indenter [16].

Although simple, this measure is error-prone, as it is obtained manually. Moreover, this task is tedious, repetitive, time-consuming, and results vary depending on the measurement criteria of each observer. Therefore, several approaches have been proposed to make its evaluation based on image processing and machine learning techniques. Sugimoto and Kawaguchi proposed an automatic process to determine the Vickers hardness of three types of samples, specular, etched specular, and rough finishes, using a method for the determination of indentation edges and corners based on statistical moments, obtaining the hardness measurement with a variable mean tolerance value depending on the applied force, being 4% when the applied force varies between 500 and 1000 g-force [17]. Dominguez and Wiederhold implemented an algorithm in which the image is binarized based on its mean value, regardless of the shape of the histogram and determines the vertices of the slit, using the Harris–Stephen corner detection method. The lengths of the diagonals are then calculated to determine the Vickers hardness number, obtaining a maximum error of 6% [18]. Fedotkin et al. proposed a method based on the comparison of the optical properties inside and outside the indentation, for which a series of circles are generated that allow information to be obtained from the histogram and the Hue distributions inside and outside them. Subsequently, the circle that is closest to the indentation area is selected, the value of the diagonals is determined and subsequently the value of the hardness, presenting 95% confidence intervals [19]. On the other hand, Privezentsev et al. developed a method to estimate a hardness value using neural networks, concluding that image processing techniques should be combined with neural networks to obtain better results [20]. Likewise, Tanaka et al. implemented an automatic method based on convolutional neural networks (CNN) using as inputs images with ideal indentation, surface roughness, distorted indentations, and cracks to measure indentation diagonals and Vickers hardness automatically and robustly, obtaining a maximum error of 6% according to the reported results [21]. Jalilian and Uhl implemented deep learning techniques from a fully convolutional neural network (FCN) to locate, segment the indentation trace, determine the position and value of the diagonals, and subsequently obtain the hardness value, presenting high robustness to the size, location, and rotation of the indentation print, as well as the brightness and surface defects contained in the image [22]. Li and Yin implemented CNN to segment the indentation footprint of the image background, and, in turn, use a bounding box to measure the length of the diagonals and determine the value of hardness in different materials, showing maximum relative errors for the diagonals length of 0.39% for TiO_2_, 1.67% Cu and 1.64% Nylon [23]. Cheng et al. used indentation images of medium carbon chrome-molybdenum steel alloys (SCM 440) with revealed microstructure, making it difficult to see the indentation trace. In addition, they implemented convolutional neural networks with different backbones used to extract different characteristics, obtaining as a result the hardness value, presenting an absolute error of about 10.2 [24].

Although machine learning techniques such as neural networks have been implemented, they require a large amount of images for training and a high computational cost for processing. In general, hardness estimation using image processing and machine learning approaches presents difficulties in application when the indentation trace is not well defined or noisy. Therefore, this paper proposes a new indentation measurement method that combines three different solutions, allowing for obtaining a higher accuracy, reducing the analysis time and achieving reproducible results. The method was implemented as a plugin of the ImageJ software, allowing for determining the Vickers hardness of the indentation mark of D2 steel.

## 2. Materials and Methods

### 2.1. Materials

**Figure 2:**
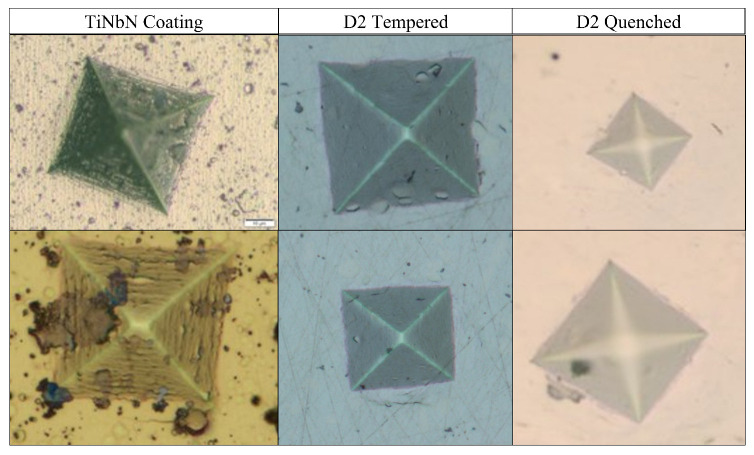
Example of indentation images used for the development of the proposed technique.

For the development of this work, 28 color images with different sizes of D2 steel in initial state, quenching, tempering heat treatment, and TiNbN coating were acquired with an optical microscope, as illustrated in Figure 2.

An expert obtained the hardness value of each sample by zooming in on the image to determine the corners of the indentation more accurately. These values were used as a golden standard for the validation of the proposed method.

For the development of the application, an Intel Core (TM) i5 8300H CPU @ 2.30 GHz computer with 12 GB RAM, running on the x64 bits Windows 10 platform, was employed. The method was implemented in Java language as a plugin of the free access application ImageJ.

### 2.2. Methods

**Figure 3:**
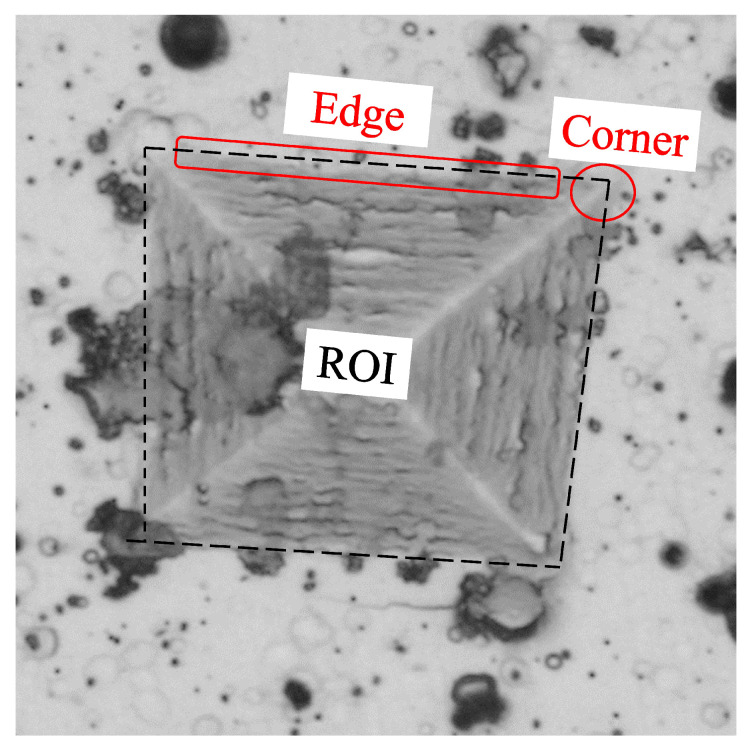
Details of the indentation image that affect the hardness measurement. Corner and edge defects and background noise.

**Figure 4:**
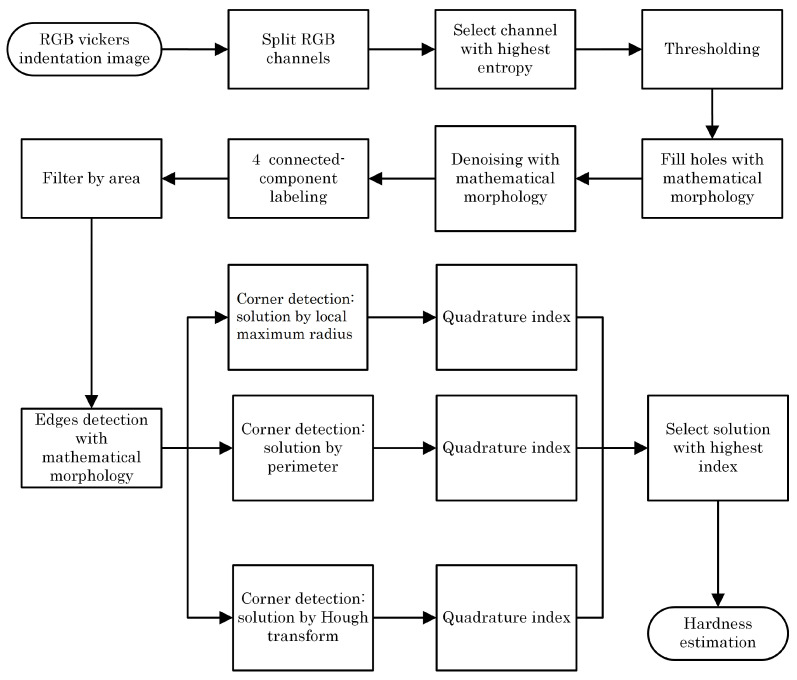
Flowchart.

As shown in Figure 3, the indentation images have poorly defined edges and corners. In addition, there are elements (noise) in the background that do not correspond to the object of interest, which affects its detection, so the result depends on the perspective of the observer. Therefore, to improve accuracy and reduce measurement time and result variation, a method based on image processing and computer vision is proposed that automatically detects indentation and estimates the Vickers hardness value. The flowchart describing the process is shown in Figure 4.

The corresponding algorithm works, in essence, as shown in the pseudocode below, obtaining the indentation region using thresholding in the color channel that has maximum entropy. Then, the corners of the indentation are obtained using three different methods, and the best one is selected according to the index proposed in this work as follows:
**Pseudocode****Input:**            Color image Q**1. Find indentation mark**            Get color channels R, G, B from Q            Find channel A with Maximun entropy            Binarize A, using the proposed method            Fill holes and remove noise using mathematical morphology            Label objects            Select larger object**2. Indentation corner detection:**             Using local maximum radius:                          Find corners $C_{R}$                         Get Quadrature index $Q_{R}$             Using indentation perimeter:                         Find corners $C_{P}$                         Get Quadrature index $Q_{P}$             Using Hough transform:                         Find corners $C_{H}$                         Get Quadrature index $Q_{H}$**3. Find best indentation result:**            if ($Q_{R}+0.05$ > $Q_{P}$ && $Q_{R}+0.05$ > $Q_{H}$) {                        Select local maximum radius solution
}            else if ($Q_{P}+0.05$ > $Q_{H}$ && $Q_{P}+0.05$ > $Q_{R}$) {                        Select Perimeter solution
}            else{                        Select Hough solution
}**4. Calculate hardness estimation HV.****Output: HV**

Since color does not provide relevant information for the study of microindentation, the channel with the highest entropy was chosen as it contains the most information for further processing.

Once the channel to be processed is selected, the region of interest (ROI) is separated from the background. As can be seen, the indentation area has a darker color with respect to the surrounding background. Therefore, the use of a thresholding method for segmentation is appropriate.

**Figure 5:**
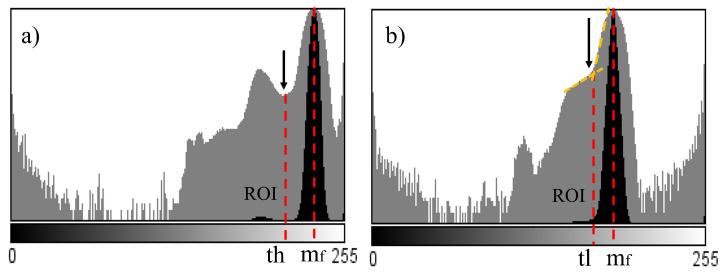
Histogram (**a**) bimodal, find the minimum point; (**b**) modal, find the change in slope.

**Figure 6:**
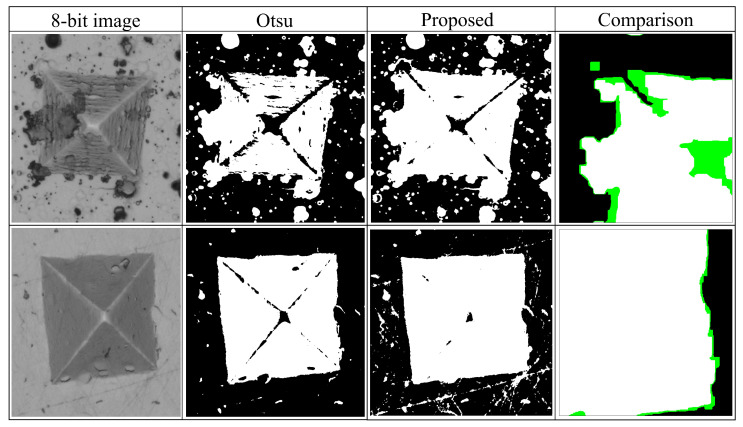
Threshold techniques evaluation. The comparison section shows Otsu (in white) and the proposed method (in green) after the filter by area process.

However, since the size of the ROI is small with respect to the image background and the gray levels between the background and the ROI are similar, the corresponding mode of the ROI is not clearly defined (see Figure 5). Therefore, the use of classical thresholding techniques such as Otsu or maximum entropy are not appropriate in this case, as they do not always give correct segmentation results (see Figure 6).

Hence, a new thresholding method was developed based on the histogram characteristics. As can be seen, the mode corresponding to the ROI is next to the one corresponding to the background and is not always distinguishable. Therefore, the following thresholding procedure was designed.

**Figure 7:**
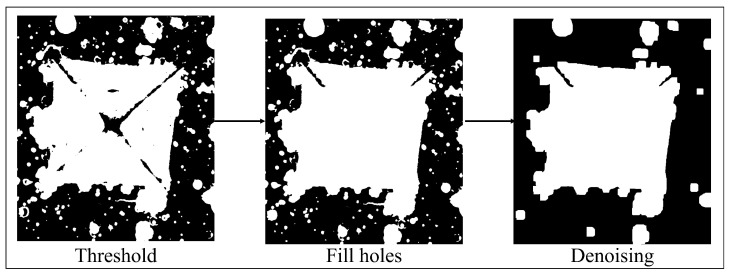
Thresholding, fill holes, and denoising.

Initially, the highest value of the histogram is searched for, which allows for identifying the mode ($m_{f}$) corresponding to the background. Since the mode corresponding to the ROI is below $m_{f}$, and is distinguished from $m_{f}$ by a local minimum that appears between them, this value is identified as the upper threshold (th) of the mode (see Figure 5a). In other histograms, the ROI is much smaller and no mode corresponding to it appears. In this case, the threshold is detected by the change of slope, as shown in Figure 5b. The low threshold (tl) is located at the point where a new slope change is found. Once the image has been segmented, some holes are visible in the ROI, to fill them, a mathematical morphology method called fill holes is used (see Figure 7).

Due to the fact that heat treatment processes were applied to the material, the image shows stains in the indentation zone, which translates into noise or unnecessary elements in the zone of interest (ROI). In order to reduce this noise, mathematical morphology techniques based on erosion and dilation operations were used, performing an opening process in order to obtain the lowest possible noise without modifying the shape and size of the indentation mark as seen in Figure 7.

**Figure 8:**
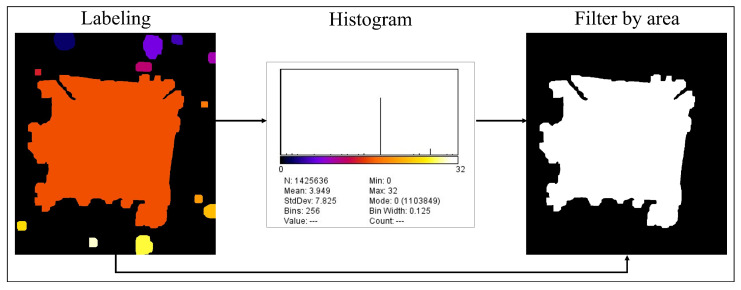
Label and area filter.

Since the indented area corresponds to the largest object, to obtain the indentation trace, all objects were labeled and the largest one, i.e., that composed of the highest number of pixels, was selected, as seen in Figure 8.

**Figure 9:**
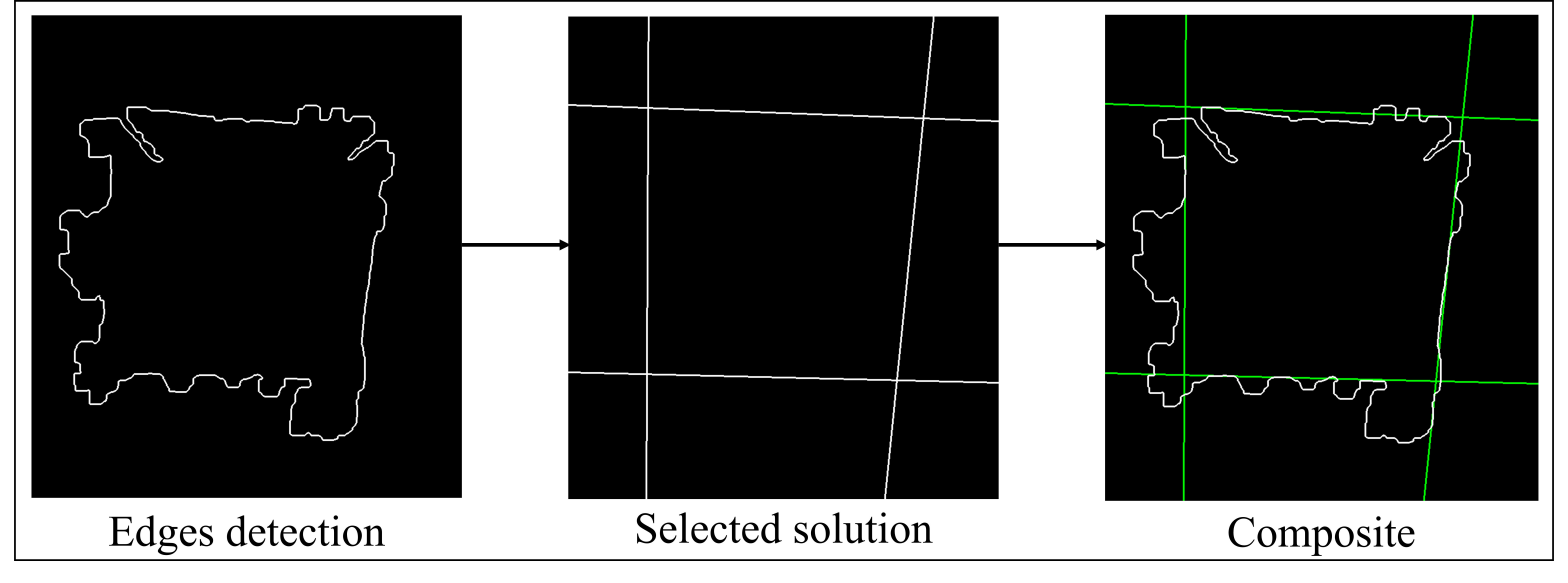
Edge detection and solution selected by the algorithm.

In order to evaluate the shape of the indentation mark, it is necessary to obtain the edges of the figure. For this purpose, mathematical morphology techniques are used by performing the difference between the image filtered by area, and this same image but eroded by one pixel. (see Figure 9 first section).

The indentation mark can take different shapes depending on the type of material and the force exerted on it by the indenter. Due to this, in many cases, it is not possible to approximate the shape of the indentation to a square. Therefore, three solutions are proposed to detect the corners of the indentation:

(a) Solution by local maximum radius: This solution works in the case where the shape of the indentation trace can be approximated to a square. If this is the case, the centroid of the figure is calculated and the four major diagonals are found, which would correspond to the four corners. In this solution, the distances of the pixels of the perimeter to the centroid of the figure are found, in such a way that, to find the corners, it is taken into account that their distance is a local maximum in the function of distances with respect to the centroid. This strategy is a rather simplified version of the method used to recognize figures from the distances relative to the centroid [25].
(b) Perimeter solution: In the case that the region of interest is affected by “noise” that may be due to the heat treatment applied or the type of material, this solution would be useful. For this solution, we calculate the perimeter of the figure, and perform a linear regression using least squares for each side. The intersections of these regressions would correspond to the four corners of the indentation mark. If the edge of the figure is “broken” or open, there would be the disadvantage of an infinite diagonal, which makes it impossible to calculate the perimeter of the figure.
(c) Solution by Hough transform: this solution has the advantage that the pixels do not need to be contiguous to determine a line; therefore, it favors the detection under a certain level of noise. It also does not limit us to obtain a single solution as in the case of a linear regression; with this transform, multiple lines can be drawn by adjusting to the irregularity of the object of interest [26].

**Figure 10:**
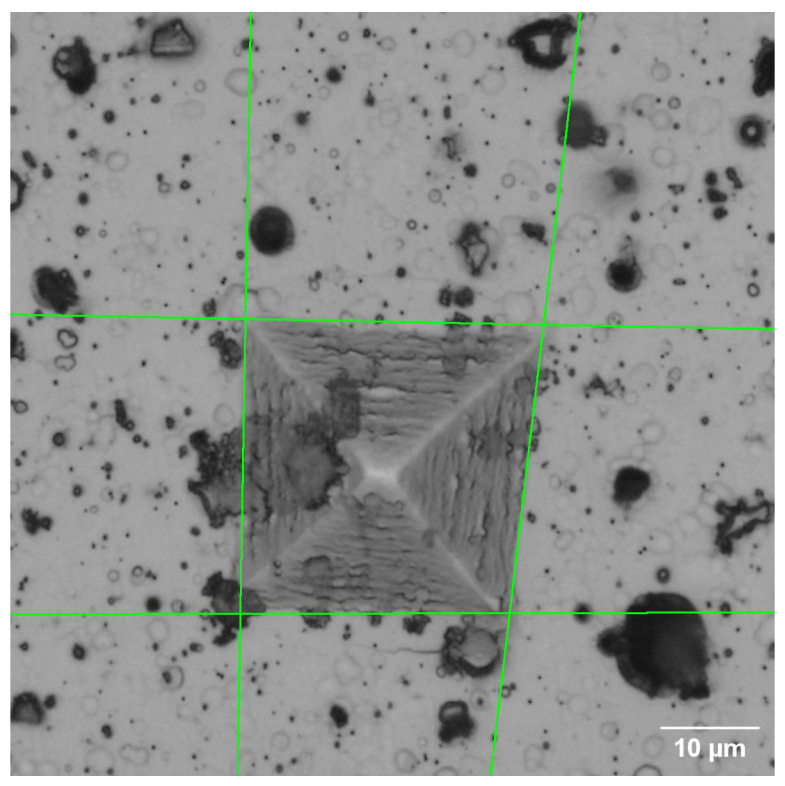
Result of corner detection.

To select the best solution (see Figure 10), the quadrature index (*Q*) given by Equation (2) was used. The proposed index allows for quantifying from the coordinates of four corners their correspondence to a square; this is achieved by taking a value between one and zero, being one for the case of an approximation to a perfect square, and decreasing towards zero as it moves away from the ideal case. Two normalized error coefficients between zero and one, Maximum Absulute Error (MaAE) and Dice Coefficient (DC), are used to take into account two characteristics of a square, the perimeter and area, respectively:

(2) $$Q=1-\frac{MaAE+DC}{2}$$

(3) $$MaAE=\max_{i=1}^{4}\frac{|s_{i}-\bar{s}|}{\bar{s}}$$

(4) $$DC=\frac{2|s_{a}-\bar{s}|}{s_{a}+\bar{s}}$$

The MaAE arises as a measure of the maximum absolute error normalized to Equation (3). Knowing the coordinates of the four corners, the distance from each side, $s_{i}$, and the average side, $\bar{s}$, are calculated. In this way, the value of MaAE reflects the maximum error encountered when estimating the sides as the perimeter divided into four parts. If all sides are equal, the error given by MaAE is zero, although this does not guarantee that it is indeed a square; for this reason, the estimation of the sides taking into account the area is also taken into account. The coefficient DC is proposed as a measure of the error associated with the estimation of the sides assuming that the area corresponds to a square; such side is denoted as $s_{a}$, and is equal to the square root of the area defined by the four corners. Formula (4) is used to calculate DC, which is based on the Dice Score formula.

## 3. Results and Discussion

Table 1 and Table 2 show the Vickers hardness results obtained manually (*M*) and with the proposed method (*A*), as well as the Error Rate, defined in Equation (5) and execution time of the algorithm to determine the corners of the indentation mark selected by the technique. To choose the best corner detection solution, the highest quadrature index is selected. In order to improve the accuracy in the selection of these solutions, a threshold of 0.05 is set for the cases in which the values of these indices are close to each other. For one of the solutions to be selected, its index value must be at least 0.05 above the other two quadrature indices. If this condition is not met, the Maximum Local Radius solution ($Q_{R}$) is given priority for selection:

(5) $$\mathrm{Error}\;\mathrm{Rate}=\frac{\left|M-A\right|}{M}*100$$

As shown in Table 1 and Table 2, the proposed method presents an error between 0.21% and 4.45% when using the algorithm based on the local maximum radius, between 1.38% and 2.87% for the perimeter solution, and between 0.02% and 4.00% for the Hough transform one.

As already mentioned, manual measurement is prone to errors because the results are variable and depend on the observer, the condition of the samples, the difficulty in detecting corners, and the number of measurements to be performed. The time required to determine the hardness manually is an estimated 4 min per sample, including the identification and measurement of the diagonals under the microscope and the determination of the hardness. The proposed method reduces the measurement time, has no variable results, and can be adapted to perform multiple measurements from the images of the indentation marks. The proposed thresholding method allows for good indentation region separation to be obtained in most cases, but it can fail if the acquisition protocol used is inadequate, producing poorly focused images, shadows, or the region of interest being too small with respect to the background.

**Figure 11:**
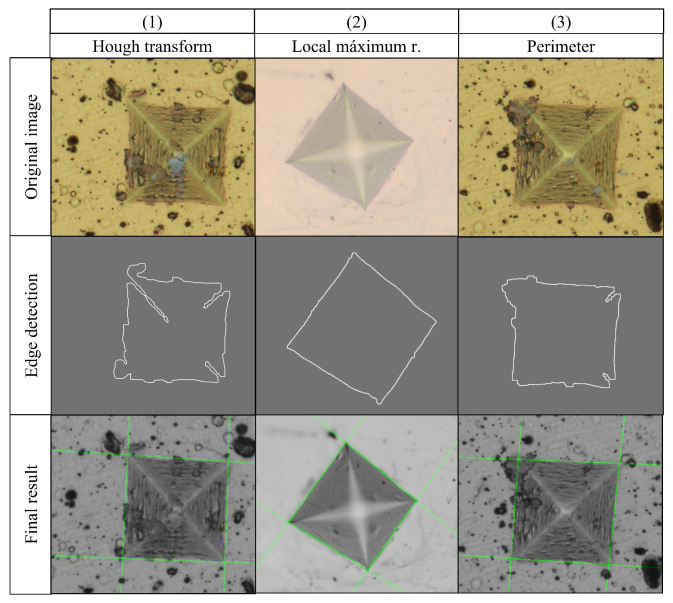
Example of proposed solutions. Column 1. Sample TiNbN-2; Column 2. Sample Steel-3; and Column 3. Sample TiNbN-3 with applied force of 10N.

Figure 11 illustrates the cases for which one of the three solutions works. For the first case, when the edge detection processing is performed, we notice that a rather irregular figure is obtained since the illumination gradient is taken into account when thresholding, which does not allow for defining the corners correctly. For this case, the solution by local maximum radius would be imprecise and also the solution by perimeter since, if it is evaluated from the centroid of the figure, it would take into account the deformities within it.

The second case is the ideal case, since the figure can be very close to a square, so this is the solution automatically chosen by the algorithm.

In the third case, it is observed that in one of the corners there is a small deformation due to the characteristics of the material and its coating. If evaluated with the solution by local maximum radii, the end of this deformation would be taken as a local maximum (see section on edge detection) since it is evaluated from the centroid of the figure, which makes the calculation of the hardness of the material imprecise. If the Hough transform is used instead, more processing time will be required.

Despite the fact that the images contain an irregular surface such as pores, scratches or microdroplets, the algorithm did not present any problems in determining the hardness of the material (see Appendix A), which shows the results obtained, thus validating its high robustness. Compared to other methods, these have been developed with marks that by their shape can be approximated to a square; this one proposes three different solutions depending on the characteristics of the figure given by the indentation mark.

The execution time for the tested images was an average of 2.05 s, taking into account that this time varies depending on the selected solution and the resolution of each image.

## 4. Conclusions

In this work, a new automatic method based on image processing is proposed to determine the Vickers hardness of AISI D2 steel in different thermal conditions such as: steel with and without quenching, tempering heat treatment and samples coated with titanium niobium nitride (TiNbN). The algorithm includes three options to determine the corners of the indentation pattern by automatically selecting the best one according to the calculated squareness indices, which allows for obtaining good results in the presence of background noise such as spots or pores on the surface and irregularities present in the indentation pattern. The proposed method allows for obtaining reproducible Vickers hardness results in an average time of 2.05 s with an accuracy of 98.3% and a maximum error of 4.5% with respect to the values measured manually, used as a gold standard, surpassing the results achieved in other previously published works.

## Figures and Tables

**Table 1:** Vickers hardness results with 5N applied force, where “Steel” corresponds to steel in its initial state, $Q_{H}$ to the quadrature index of the solution by Hough, $Q_{P}$ to the quadrature index of the solution by Perimeter and $Q_{R}$ to the quadrature index of the Local Maximum Radius.

Material	Algorithm (HV)	Manual (HV)	Error Rate	Runtime (s)	$Q_{H}$	$Q_{P}$	$Q_{R}$	Selected Solution
Tempered-1	620.46	625.49	0.80	1.39	0.971	0.979	0.975	Max radius
Tempered-2	609.32	610.61	0.21	1.42	0.981	0.981	0.980	Max radius
Tempered-3	632.88	626.54	1.01	1.40	0.968	0.178	0.970	Max radius
TiNbN-1	1105.00	1104.74	0.02	1.34	0.978	0.423	0.926	Hough
TiNbN-2	1125.77	1172.74	4.00	1.36	0.989	0.934	0.939	Hough
TiNbN-3	1127.42	1157.46	2.60	0.83	0.984	0.413	0.928	Hough
TiNbN-4	1122.60	1150.30	2.41	0.97	0.983	0.087	0.497	Hough
Steel-1	174.54	168.70	3.46	4.74	0.922	0.949	0.955	Max radius
Steel-2	169.71	164.59	3.11	4.72	0.947	0.954	0.959	Max radius
Steel-3	177.11	172.65	2.58	4.76	0.963	0.961	0.965	Max radius
Quenched-1	742.65	711.03	4.45	1.99	0.954	0.972	0.970	Max radius
Quenched-2	659.97	640.88	2.98	1.14	0.959	0.966	0.969	Max radius
Quenched-3	628.92	620.36	1.38	1.61	0.598	0.961	0.822	Perimeter

**Table 2:** Vickers hardness results with 10N applied force, where “Steel” corresponds to steel in its initial state, $Q_{H}$ to the quadrature index of the solution by Hough, $Q_{P}$ to the quadrature index of the solution by Perimeter and $Q_{R}$ to the quadrature index of the Local Maximum Radius.

Material	Algorithm (HV)	Manual (HV)	Error Rate	Runtime (s)	$Q_{H}$	$Q_{P}$	$Q_{R}$	Selected Solution
Tempered-1	604.27	599.50	0.80	0.93	0.971	0.979	0.980	Max radius
Tempered-2	612.89	609.86	0.50	0.92	0.981	0.973	0.974	Max radius
Tempered-3	593.55	595.47	0.32	0.89	0.974	0.975	0.977	Max radius
TiNbN-1	1060.26	1075.99	1.46	0.74	0.973	0.970	0.969	Max radius
TiNbN-2	1074.71	1069.96	0.44	0.86	0.991	0.678	0.551	Hough
TiNbN-3	1145.3	1113.35	2.87	0.89	0.982	0.990	0.914	Perimeter
TiNbN-4	950.06	931.23	2.02	0.96	0.933	0.895	0.977	Max radius
TiNbN-5	899.22	910.07	1.19	0.97	0.984	0.879	0.930	Hough
TiNbN-6	917.31	908.14	1.01	0.90	0.983	0.518	0.874	Hough
Steel-1	267.16	261.84	2.03	4.74	0.970	0.971	0.969	Max radius
Steel-2	269.78	264.61	1.95	4.73	0.969	0.958	0.962	Max radius
Steel-3	273.06	271.75	0.48	4.86	0.945	0.958	0.960	Max radius
Quenched-1	1020.45	1029.43	0.87	3.55	0.968	0.982	0.962	Max radius
Quenched-2	980.49	967.21	1.37	1.70	0.953	0.968	0.969	Max radius
Quenched-3	967.41	943.70	2.51	2.26	0.954	0.964	0.966	Max radius

**Figure A1:**
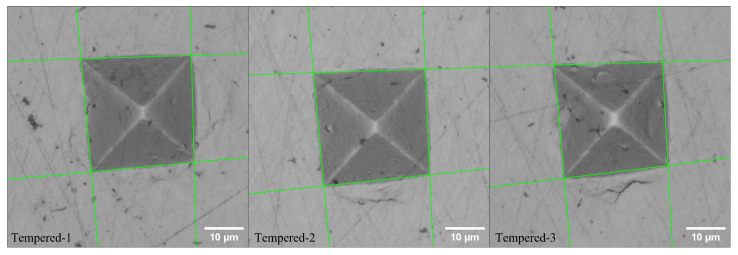
Corner detection on Tempering samples at 5N applied force.

**Figure A2:**
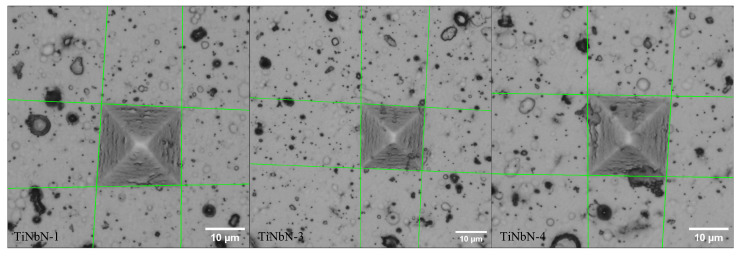
Corner detection on TiNbN samples at 5N applied force.

**Figure A3:**
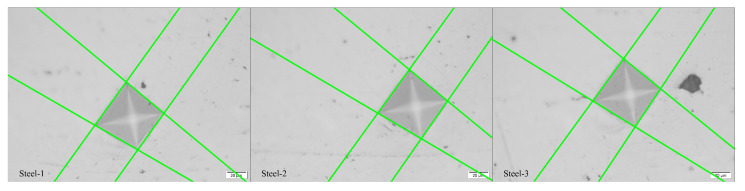
Corner detection on Steel samples at 5N applied force.

**Figure A4:**
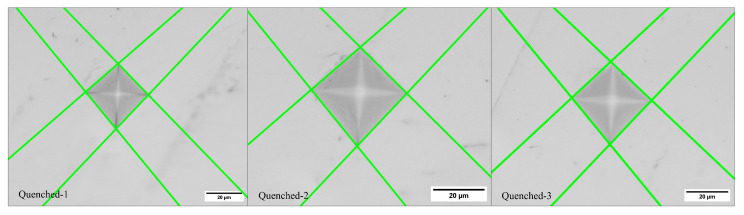
Corner detection on Quenched samples at 5N applied force.

**Figure A5:**
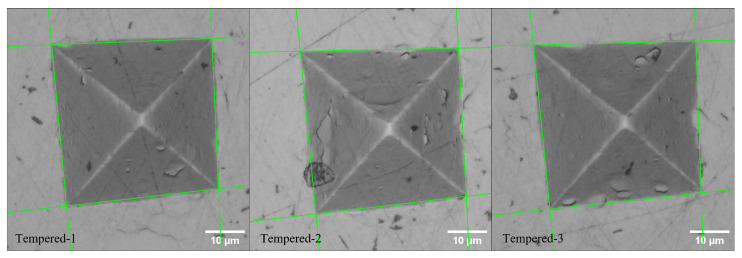
Corner detection on Tempering samples at 10N applied force.

**Figure A6:**
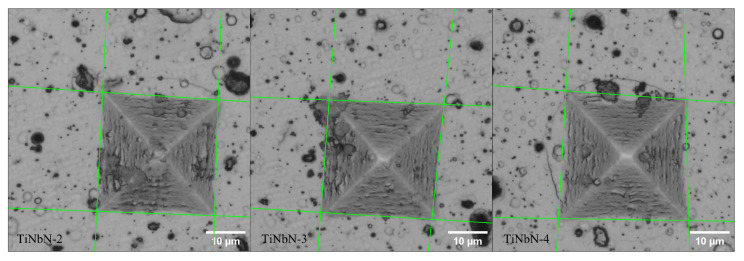
Corner detection on TiNbN samples at 10N applied force.

**Figure A7:**
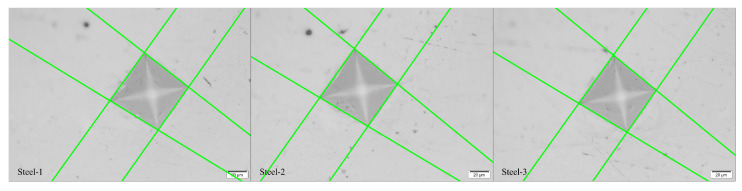
Corner detection on Steel samples at 10N applied force.

**Figure A8:**
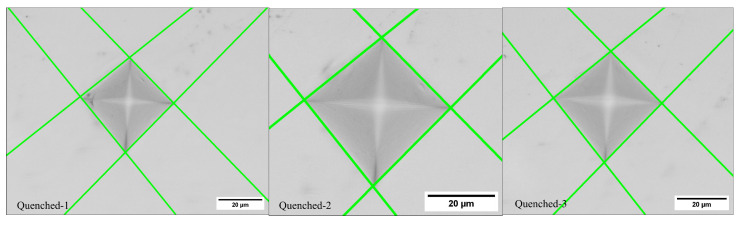
Corner detection on Quenched samples at 10N applied force.

## Data Availability

The images used for the development of this work are available at http://dx.doi.org/10.13140/RG.2.2.31695.15529, accessed on 19 December 2022. The code developed for this work, which was implemented as a plugin to the freely available ImageJ software, is freely available at: http://dx.doi.org/10.13140/RG.2.2.14917.93921, accessed on 19 December 2022.
